# Double mutation (R124H, N544S) of *TGFBI* in two sisters with combined expression of Avellino and lattice corneal dystrophies

**Published:** 2009-05-15

**Authors:** Naoyuki Yamada, Koji Kawamoto, Naoyuki Morishige, Tai-ichiro Chikama, Teruo Nishida, Mitsuaki Nishioka, Naoko Okayama, Yuji Hinoda

**Affiliations:** 1 Department of Ophthalmology, Yamaguchi University Graduate School of Medicine, Ube City, Yamaguchi, Japan; 2 Department of Ocular Pathophysiology, Yamaguchi University School of Medicine, Ube City, Yamaguchi, Japan; 3 Division of Laboratory, Yamaguchi University Hospital, Ube City, Yamaguchi, Japan

## Abstract

**Purpose:**

The R124H mutation of the keratoepithelin gene (*TGFBI*) causes Avellino corneal dystrophy whereas the N544S mutation of this same gene gives rise to lattice corneal dystrophy. We now report two cases with both R124H and N544S mutations of *TGFBI*.

**Methods:**

Genomic DNA and cDNA were isolated from the proband and family members and were subjected to polymerase chain reaction–mediated amplification of exons 1–17 of *TGFBI*. The amplification products were directly sequenced. Allele-specific cloning and sequencing were applied to evaluate the compound heterozygous mutation.

**Results:**

Molecular genetic analysis revealed that the proband and one sister harbored both a heterozygous CGC→CAC (Arg→His) mutation at codon 124 and a heterozygous AAT→AGT (Asn→Ser) mutation at codon 544 of *TGFBI*. Slit-lamp examination revealed multiple granular regions of opacity and lattice lines in the corneal stroma of the proband and her sister with the double mutation. Allele-specific cloning and sequencing revealed that the R124H and N544S mutations are on different chromosomes.

**Conclusions:**

As far as we are aware, this is the first report of a patient with a double mutation (R124H, N544S) of *TGFBI* causing an autosomal dominant form of corneal dystrophy. The clinical manifestations of the two cases with both R124H and N544S mutations appeared to be a summation of Avellino and lattice corneal dystrophies.

## Introduction

Mutations of the keratoepithelin gene (*TGFBI*) are responsible for most corneal dystrophies. *TGFBI* was first identified as a transforming growth factor-β1 (TGF-β1)-inducible gene in a human lung adenocarcinoma cell line [1]. The point mutations R124C, R124H, R555W, and R555Q of *TGFBI* were initially found to give rise to lattice corneal dystrophy (LCD), Avellino corneal dystrophy (ACD), Groenouw type I corneal dystrophy, and Reis-Bücklers corneal dystrophy, respectively [2]. Many additional mutations of *TGFBI* were subsequently found to be responsible for autosomal dominant corneal dystrophies [3,4]. ACD is characterized by the presence of granular and linear opacities in the corneal stroma. The deposits in the corneal stroma of patients with ACD are of a hyaline and amyloid nature. The only identified mutation associated with ACD is R124H of *TGFBI* [2]. LCD is an inherited form of amyloidosis that is characterized by the development of lattice lines and opacities in the cornea. Several distinct mutations of *TGFBI* including R124C [2], L518P [5], P501T [6], L527R [7], N544S [8], A546T [9], and N622K (T1913G or T1913A) [3] have been associated with LCD. LCD is classified clinically into several subtypes [3,4], but standardized definitions of each subtype have not been achieved to date. The subtype of LCD caused by the N544S mutation of *TGFBI* is characterized by tiny nodular deposits with thin lattice lines in the middle portion of the corneal stroma [10].

Several case reports have suggested that corneal dystrophies caused by homozygous point mutations of *TGFBI* are characterized by an earlier onset, more severe symptoms, and a higher frequency of recurrence after keratoplasty compared with those attributable to the corresponding heterozygous mutations [11-15]. A few case reports have also described individuals with corneal dystrophy who harbor two distinct mutations in *TGFBI*, the membrane component, chromosome 1, surface maker 1 (*M1S1*), or both [16-21]. It has remained unclear, however, how the phenotype of patients with such a double mutation differs from that of those with the corresponding single mutations. We now describe the first cases of corneal dystrophy associated with both R124H and N544S mutations of *TGFBI*.

## Methods

This study was approved by the ethical review committee for gene analysis research of Yamaguchi University School of Medicine and Yamaguchi University Hospital. After obtaining informed written consent, we extracted genomic DNA from white blood cells of peripheral blood collected from patients in the presence of an anticoagulant. Total RNA was also extracted from the white blood cells with the use of a QIAmp RNA Blood mini kit (Qiagen, Valencia, CA) and was then subjected to reverse transcription with the use of TaqMan Reverse Transcription Reagents (Applied Biosystems, Foster City, CA). The resulting cDNA as well as genomic DNA were subjected to polymerase chain reaction (PCR) with primers that amplify exons 1, 4, 11, 12, 13, 14, 2–9, or 9–17 of *TGFBI* (Table 1). Each PCR reaction was performed in a total volume of 10 μl containing template DNA (80 ng/μl), 10 pmol of each primer, 200 μM of each deoxynucleoside triphosphate, 20 mM MgCl_2_, 20 mM Tris-HCl (pH 8.0), 100 mM KCl, and 1 U of Taq polymerase (Ex Taq; Takara, Tokyo, Japan). The reaction mixture was overlaid with 10 μl of mineral oil, and amplification was performed with a Gene Amp PCR System PC808 (ASTEC, Tokyo, Japan) with an initial denaturation at 95 °C for 2 min followed by 30 cycles of denaturation at 94 °C for 30 s, annealing at 58 °C, 60 °C, or 62 °C (Table 1) for 20 s, and extension at 72 °C for 30 s. The PCR products were separated by electrophoresis on a 2% agarose gel and stained with ethidium bromide. For sequencing, 2.5 µl of the PCR products were incubated with 1 μl of ExoSAP-IT (Amersham Bioscience, Tokyo, Japan) first for 20 min at 37 °C and then for another 20 min at 80 °C. Sequencing reactions were then performed with the use of a BigDye Terminator Cycle Sequencing FS Ready Reaction Kit (Applied Biosystems). After purification with ethanol, the reaction products were applied to an ABI 3100-Avant Genetic Analyzer (Applied Biosystems).

**Table 1:** PCR primers used for sequencing exons of *TGFBI*.

**Exon**	**Primer**	**Primer sequence**	**Annealing temperature (°C)**	**Product size (bp)**
2–9	cDNA-F1	5'-CGCCAAGTCGCCCTACCAG-3'	60	1205
	cDNA-R1	5'-TTGGAGGGGTTCCATCTTTG-3'		
9–17	cDNA-F2	5'-CTCATCCCAGACTCAGCCAA-3'	60	1075
	cDNA-R2	5'-CACATCTCATTATGGTGCGGC-3'		
1	DNA-1F	5'-CCGCTCGCAGCTTACTTAAC-3'	60	362
	DNA-1R	5'-AGCGCTCCAATGCTGCAAGGT-3'		
4	DNA-4F	5'-CGTCCTCTCCACCTGTAGAT-3'	62	350
	DNA-4R	5'-GACTCCCATTCATCATGCCC-3'		
11	DNA-11F	5'-CAGCCTTAATAACCCATCCCA-3'	58	375
	DNA-11R	5'-AATCCCCAAGGTAGAAGAAAG-3'		
12	DNA-12F	5'-AGGAAAATACCTCTCAGCGTGG-3'	60	293
	DNA-12R	5'-ATGTGCCAACTGTTTGCTGC-3'		
13	DNA-13F	5'-GGGAGTTCTTCATTTCAGGG-3'	58	365
	DNA-13R	5'-ATTACACTCAGAGATTCGGG-3'		
14	DNA-14F	5'-GCCTGGGCGACAAGATTGA-3'	58	419
	DNA-14R	5'-CCAACAGCTCCCAATTCAC-3'		

An allele-specific cloning and sequencing approach was applied to characterize the compound heterozygous mutation of R124H and N544S. In brief, cDNA of the proband was subjected to PCR with KOD FX DNA polymerase (Toyobo, Tokyo, Japan) and with the primers, 5′-TGT CCA GCA GCC CTA CCA CTC-3′ (forward) and 5′-AGG ATA TCC CCT CTT TCC TGA GGT C-3′ (reverse; containing an EcoRV restriction site at its 5′ end), to obtain products that included both mutation sites. The PCR products were purified by electrophoresis and digested with EcoRV and BamHI (site in exon 4), and the released fragments were ligated into the multiple cloning site of a sequencing vector (pcDNA3.1[+]; Promega, Madison, WI). The resulting plasmids were expanded in competent *Escherichia coli* JM109 cells (Invitrogen, Carlsbad, CA), and the inserts were then sequenced as described above.

## Results

**Figure 1:**
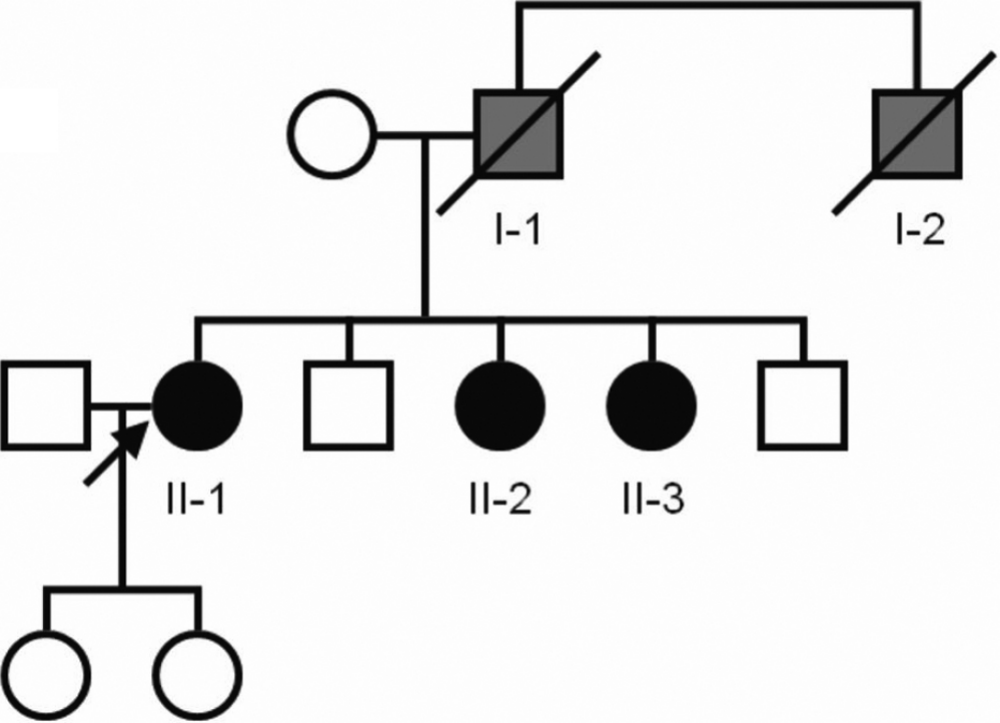
Pedigree of the proband. Black symbols indicate individuals with a diagnosis of corneal dystrophy by genetic analysis. Gray symbols indicate individuals suspected of having been affected by corneal dystrophy but not subjected to genetic analysis. The arrow indicates the proband.

The proband, a 67-year-old Japanese woman (II-1), visited our corneal clinic in January 2000 with a main complaint of gradual impairment of vision (Figure 1). We diagnosed her condition as ACD on the basis of slit-lamp examination. Given that her visual acuity had decreased to 0.7 in the right eye and 0.4 in the left eye, we performed phototherapeutic keratectomy on her left eye in March 2000 and on her right eye in May 2000. The parents of II-1 were not related to each other. Her father (I-1) is no longer alive, and she has two brothers and two sisters. Her father’s brother (I-2) and her sisters (II-2, II-3) were also diagnosed at our clinic with ACD by slit-lamp examination. Her reporting suggested that her father (I-1) had corneal dystrophy. We also performed phototherapeutic keratectomy on the left eye of II-2 in March 2000 and on the right eye of II-2 in May 2000.

**Figure 2:**
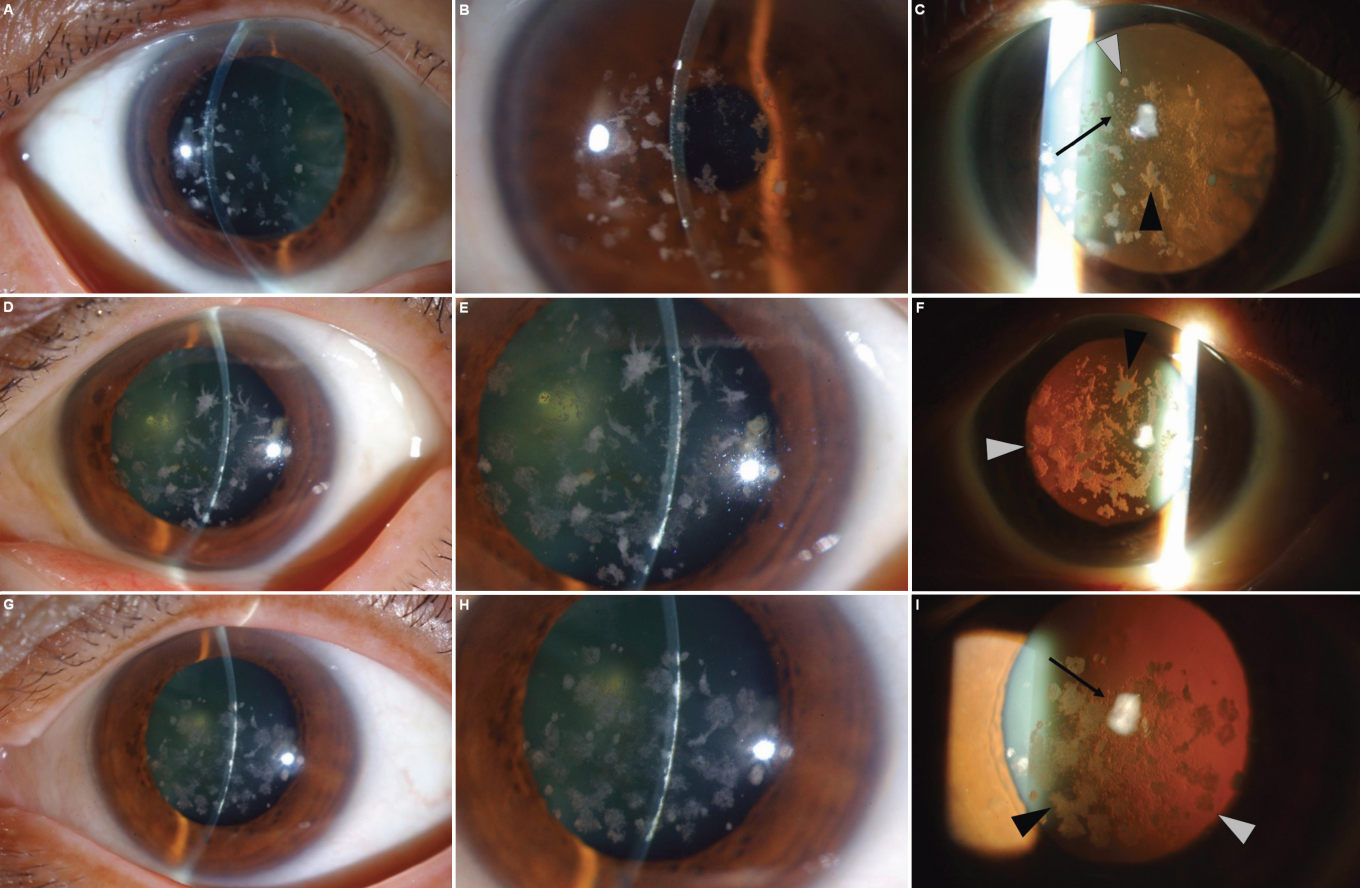
Slit-lamp photographs of the proband and her two sisters. Slit-lamp photographs of the right eye of II-1 (**A**–**C**), the left eye of II-2 (**D**–**F**), and the left eye of II-3 (**G**–**I**) are shown. Granular deposits (gray arrowheads) and star-shaped deposits (black arrowheads) were observed in all three patients (**C**,**F**,**I**) whereas thin lattice lines (black arrows) were observed only in II-1 (**C**) and II-3 (**I**). Nodular deposits were apparent mostly in the superficial-to-middle portion of the corneal stroma in all three patients (**B**,**E**,**H**).

**Figure 3:**
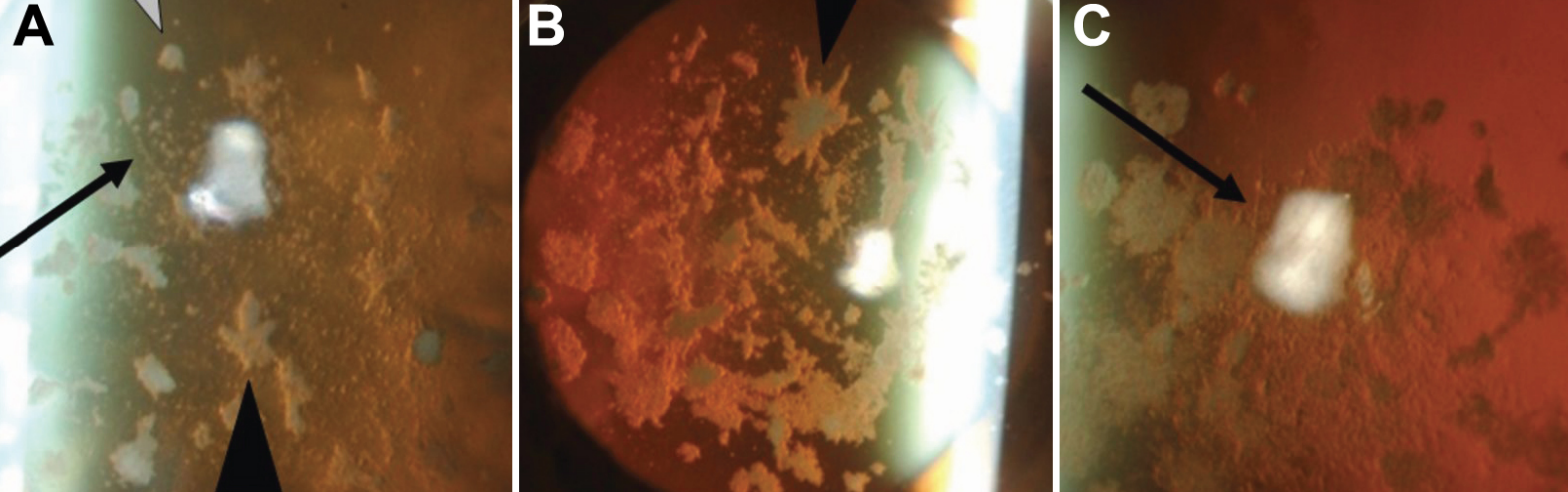
Lattice lines in patient's cornea. The lattice lines referred to in Figure 2 are better visualized in the higher magnifications of Figure 2**C**, Figure 2**F**, and Figure 2**I** (Figure 3**A**-**C**, respectively). The lattice lines are easily seen in **A** and **C** (black arrows), but not in **B**.

**Figure 4:**
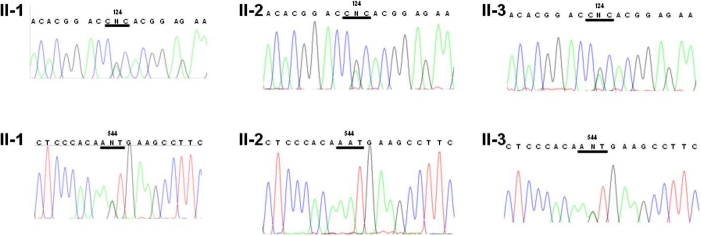
Genetic analysis of *TGFBI* in the proband and her two sisters. Direct sequencing of genomic amplification products corresponding to exon 4 (upper panels) or exon 12 (lower panels) of *TGFBI* was performed for II-1, II-2, and II-3. A heterozygous CGC→CAC mutation was detected at codon 124 in II-1, II-2, and II-3. A heterozygous AAT→AGT mutation was detected at codon 544 in II-1 and II-3.

Slit-lamp examination subsequently revealed multiple granular regions of opacity in the surface-to-middle portion of the corneal stroma in both eyes of II-1, II-2, and II-3. Lattice lines were also observed in II-1 (Figure 2A–C) and II-3 (Figure 2G–I) but not in II-2 (Figure 2D–F). These lattice lines can be seen better in the higher magnifications of Figure 2C, Figure 2F and Figure 2I (Figure 3A-C, respectively). Both II-1 and II-3 were found to harbor both a heterozygous CGC→CAC (Arg→His) mutation at codon 124 and a heterozygous AAT→AGT (Asn→Ser) mutation at codon 544 of *TGFBI* whereas II-2 harbored only the heterozygous CGC→CAC (Arg→His) mutation at codon 124 (Figure 4). The mutations were identified at both the genomic and cDNA levels.

To investigate whether the two *TGFBI* mutations are on the same or different chromosomes of the proband, we adopted an allele-specific cloning and sequencing approach. PCR products containing both mutation sites were subcloned and sequenced. Of three independent clones analyzed, one contained only the R124H mutation and the other two contained only the N544S mutation, indicating that the two mutations are on different chromosomes.

## Discussion

As far as we are aware, this is the first report of a patient with a double mutation of *TGFBI* causing an autosomal dominant form of corneal dystrophy. The clinical manifestations of the two cases with both R124H and N544S mutations appeared to be a summation of those of Avellino and lattice corneal dystrophies. We observed lattice lines in the corneas of II-1 and II-3, both of whom have the N544S mutation of *TGFBI*, but not in II-2, who harbors only the R124H mutation.

We were not able to perform genetic analysis on I-1 and I-2 because they were no longer alive at the time of this analysis. However, allele-specific cloning and sequencing revealed that the R124H and N544S mutations are on different chromosomes, consistent with our clinical findings. Slit-lamp examination of I-2 did not reveal the presence of lattice lines, suggesting that the R124H mutation was transmitted to the proband and her two sisters from I-1. Although slit-lamp examination was not performed on the mother of the three sisters because of her being confined to bed, it is likely that she harbors the N544S mutation of *TGFBI*. Given that the clinical manifestation of the N544S mutation has a late onset and that the mutation does not have a pronounced effect on visual acuity, the mother may not experience a visual disturbance.

Several cases of double mutations associated with corneal dystrophies other than macular corneal dystrophy have been described previously (Table 2). However, no case of a double mutation of *TGFBI* causing an autosomal dominant form of corneal dystrophy has previously been reported. The presence of a homozygous Q118X mutation of *M1S1* and a heterozygous P501T mutation of *TGFBI* in the same individual was described [16]. The Q118X mutation of *M1S1* causes gelatinous drop-like corneal dystrophy (GDLD) with an autosomal recessive mode of inheritance. The P501T mutation of *TGFBI* causes LCD type IIIA [6]. The clinical manifestation in this patient resembled that of GDLD but not that of LCD type IIIA. A patient with a clinical diagnosis of GDLD and heterozygous Q118X and Y184C mutations of *M1S1* has also been described [17]. No other case of the Y184C mutation in *M1S1* has been presented, so it is not clear whether this mutation in the homozygous state can cause GDLD. A patient with a clinical diagnosis of GDLD was found to be heterozygous for both Q118X and L186P mutations of *M1S1* [18]. Patients with a clinical diagnosis of atypical LCD were found to be heterozygous for both A546D and P551Q mutations of *TGFBI* [19,20]. The A546D mutation of *TGFBI* causes polymorphic corneal amyloidosis [22] or atypical LCD [23] with an autosomal dominant mode of inheritance. There have been no other reports of the P551Q mutation of *TGFBI*, so it is not clear whether a heterozygous P551Q mutation causes corneal dystrophy. Finally, a patient with a clinical diagnosis of granular corneal dystrophy was found to be heterozygous for both R124L and ΔT125-ΔE126 mutations of *TGFBI* [21]. There have been no other reports of the ΔT125-ΔE126 mutation of *TGFBI*.

**Table 2:** Previous reports of double mutations associated with corneal dystrophy.

**Case**	**Amino acid mutation**	**Hetero- or homozygote**	**Gene**	**Mode of inheritance**	**Phenotype of single mutation**	**Phenotype of double mutation**	**Reference**
1	Q118X	Homozygote	*M1S1*	AR	GDLD	GDLD	[16]
	P501T	Heterozygote	*TGFBI*	AD	LCD		
2	Q118X	Heterozygote	*M1S1*	AR	GDLD	GDLD	[17]
	Y184C	Heterozygote	*M1S1*	Not identified	Not identified		
3	Q118X	Heterozygote	*M1S1*	AR	GDLD	GDLD	[18]
	L186P	Heterozygote	*M1S1*	AR	GDLD		
4	A546D	Heterozygote	*TGFBI*	AD	Polymorphic corneal amyloidosis or LCD	LCD	[19,20]
	P551Q	Heterozygote	*TGFBI*	Not identified	Not identified		
5	R124L	Heterozygote	*TGFBI*	AD	GCD	GCD	[21]
	DeltaT125-DeltaE126	Heterozygote	*TGFBI*	Not identified	Not identified		
Present case	R124H	Heterozygote	*TGFBI*	AD	ACD	ACD+LCD	Present study
	N544S	Heterozygote	*TGFBI*	AD	LCD		

Abbreviations: ACD, Avellino corneal dystrophy; AD, autosomal dominant; AR, autosomal recessive; GCD, granular corneal dystrophy; GDLD, gelatinous droplike corneal dystrophy; LCD, lattice corneal dystrophy.

A few studies have addressed the penetrance of inherited corneal dystrophy. LCD type IIIA caused by the P501T mutation of *TGFBI* [16] and atypical granular corneal dystrophy caused by the D123H mutation of *TGFBI* [24] are thought to have a low penetrance. Non-penetrance of ACD has also been described [25]. The penetrance of corneal dystrophies caused by the R124H or N544S mutations of *TGFBI* remains unclear.

In all previously reported cases of double mutations, the clinical phenotype resembled that of one but not both of the associated corneal dystrophies. In the cases described in the present study, the phenotype associated with the double mutation is the summation of both corneal dystrophies. These cases thus indicate that R124H and N544S mutations of *TGFBI* independently determine clinical manifestation.
